# Comparison of risk factors for ischemic stroke and coronary events in a population-based cohort

**DOI:** 10.1186/s12872-021-02344-4

**Published:** 2021-11-12

**Authors:** Iram Faqir Muhammad, Yan Borné, Suneela Zaigham, Martin Söderholm, Linda Johnson, Margaretha Persson, Olle Melander, Gunnar Engström

**Affiliations:** 1 Department of Clinical Sciences, Lund University, CRC 60:13, Jan Waldenströms gata 35, 20502 Malmö, Sweden; 2 Skåne University Hospital, Malmö, Sweden

**Keywords:** Competing risk analysis, Cardiovascular disease, Epidemiology, Ischemic stroke, Risk factors

## Abstract

**Background:**

Although coronary events (CE) and ischemic stroke share many risk factors, there are also some important differences. The aim of this paper was to assess the association of risk factors in relation to incident CE and ischemic stroke and to evaluate the heterogeneity in patterns of risk factors between the two outcomes.

**Method:**

Traditional risk factors and inflammatory markers associated with coronary events and ischemic stroke were measured in the Malmö Diet and Cancer Cohort (MDCS, n = 26 519), where a total of 2270 incident ischemic stroke and 3087 incident CE occurred during a mean follow up time 19 ± 6 years, and in relation to inflammatory markers in the cardiovascular sub-cohort (MDC-CV, n = 4795). Cox regression analysis was used to obtain hazard ratios. A modified Lunn-McNeil competing risk analysis was conducted to assess the significance of any differences in risk profiles of these outcomes.

**Results:**

Most cardiovascular risk factors were associated both with incident CE and ischemic stroke. However, current smoking, ApoB, low ApoA1, male sex and education level of ≤ 9 years of schooling were preferentially associated with CE compared to ischemic stroke. Conversely, age showed a stronger association with ischemic stroke than with CE.

**Conclusion:**

CE and ischemic stroke have broadly similar risk factors profiles. However, there are some important differential associations, as well as substantial differences in the magnitude of the association. These could reflect the distinct biology of atherogenesis in different vascular beds. The difference in the determinants highlights the importance of looking at CE and ischemic stroke, two manifestations of cardiovascular disease, separately.

**Supplementary Information:**

The online version contains supplementary material available at 10.1186/s12872-021-02344-4.

## Introduction

Cardiovascular diseases (CVD) are one of the leading causes of morbidity and mortality in the world [1]. Identifying modifiable risk factors for stroke and coronary events (CE) is essential for preventive measures and treatment recommendations. Both conditions share many risk factors which have been well documented [2]. However, epidemiological studies have revealed that there are also some important differences. For instance, elevated cholesterol levels have been more strongly linked to CE than stroke [3–5]. On the other hand, hypertension has shown to be preferentially associated with incidence of stroke [6]. Furthermore, although atherogenesis in carotid and coronary vessels show many similarities, there are also differences between the vessels with respect to plaque morphology and characteristics as well as presence of perivascular fat [7].

Based on established risk markers, risk prediction models for CVD have been developed [8]. However, controversies exist and risk factors have shown differences with respect to the prediction of CE and stroke [9] 10. Therefore, breaking down the composite endpoint of CVD into CE and ischemic stroke may help in assessing these risks separately and provide additional information to improve predictability. Few studies have explored the differences in association between risk factors for these conditions [11, 12]. Establishing whether well-known risk factors contribute in a similar manner towards the risk of CE and ischemic stroke is essential for understanding underlying pathology and could be used to find hypotheses for developing interventions.

The aim of the present study was to assess the association of various traditional risk factors and inflammatory markers to the risk of ischemic conditions namely, incident CE and ischemic stroke, and to explore any heterogeneity in the strength of association between these two outcomes.

## Materials and methods

### Study population

The Malmö Diet and Cancer Study (MDCS) is a large population-based cohort with a prospective design [13]. In total, 28,449 subjects (11,246 men and 17,203 women) from the eligible population of 68,905 individuals participated in baseline examinations between 1991 and 1996. We excluded those with a history of stroke or CE (n = 841). To rule out acute inflammatory conditions and laboratory errors, those with a total leukocyte count higher than 20 × 10^9^/L were also excluded [14]. Finally, all participants with missing values for co-variates were also excluded from the final study population. Therefore, complete information regarding risk factors, leukocyte count and covariates was available for the total number of 26,519 participants. The traditional risk factors and total and differential leukocyte count were explored with relation to incident CE and ischemic stroke in this study population.

A random sample of participants from the MDCS were invited between October 1991 and February 1994 to study the epidemiology of carotid artery atherosclerosis. This sub-cohort, consisting of 6103 individuals (2572 men and 3531 women), was named the Malmö Diet and Cancer Cardiovascular cohort (MDC-CV) [15]. Seven inflammatory markers namely alpha1-antitrypsin, orosomucoid, haptoglobin, complement C3 (C3), C-reactive protein (CRP) and soluble urokinase plasminogen activator receptor (suPAR), were explored in relation to incident CE and ischemic stroke in this sub cohort. For this analysis, cases of CE and stroke, that had occurred between baseline screening and this re-examination, were excluded (n = 147) as well as those missing values for co-variates (n = 1161) resulting in a study population of 4795. Lastly, the missing number of measurements for each of the inflammatory markers were excluded in separate analyses for each inflammatory marker.

Study populations for both the cohorts are illustrated in a flow chart in Additional file 1: Fig. 1.

### Baseline measurements

The baseline measurements of the MDCS consisted of a self-administered questionnaire, anthropometric measurements and collection of non-fasting blood samples, described in detail previously [13].

Blood pressure (mmHg) was measured once, after ten minutes of rest, while the subject was in supine position, and using a mercury-column sphygmomanometer. Waist circumference (cm) was measured midway between the lowest rib margin and the iliac crest. Information regarding smoking, use of anti-hypertensive medications and education level was obtained from the questionnaire. Smoking status was dichotomized into current or former smokers and non-smokers. Education level was divided into two categories; ≤ 9 years of schooling and > 9 years of schooling (reference category). Apolipoprotein A1 (ApoA1) and apolipoprotein B (ApoB) concentrations were determined by Quest Diagnostics (San Juan Capistraon, CA, USA) in serum samples that had been stored at − 80 °C until the analysis was performed in 2013 using an immunonephelometric assay that was run on a Siemens BNII (Siemens, Newark, DE, USA) [16]. Total and differential leucocyte counts (neutrophils, lymphocytes, mixed cells) were determined in heparinized whole blood using Sysmex K 1000 (Sysmex Europe, Norderstedt, Germany) at the central laboratory of Malmö Hospital, within 2 h from blood sample collection. Diabetes mellitus (DM) at baseline was defined based on a self-reported physician’s diagnosis of DM, or use of anti-diabetic medication, and retrieved from several local and national registers, which have previously been described in detail [17].

In the MDC-CV, additional fasting blood samples were drawn. The low-density lipoprotein (LDL) concentration was calculated according to the Friedewald’s formula. The plasma levels of ceruloplasmin, orosomucoid, haptoglobin, alpha1-antitrypsin and C3 were determined using Cobas c-systems (Roche Diagnostics GmbH, Germany) [18]. High-sensitivity CRP in plasma was analyzed using the Tina-quant®CRP latex assay (Roche Diagnostics, Basel, Switzerland) on an ADVIA®1650 Chemistry System (Bayer Healthcare, NY, USA). SuPAR was determined using the commercial ELISA suPARnostic kit (ViroGates, Copenhagen, Denmark) [19].

### Outcome ascertainment

The two outcomes of interest in the study were incident CE and incident ischemic stroke. Participants were followed from baseline examination until first CE or ischemic stroke event, emigration from Sweden, death, or end of follow-up (December 31st, 2016), whichever came first.

Information regarding incident CE and ischemic stroke were obtained from local and national registers. Briefly, the Swedish Hospital Discharge Register and the National Cause of Death Register were used for CE. A CE was defined as a fatal or non-fatal myocardial infarction (International Classification of Diseases 9th (ICD-9) codes 410) or death due to ischemic heart disease (codes 411, 412 and 414 (ICD-9) or I21-25 (ICD-10)).

Up until 2010, incident stroke cases were retrieved by linkages with both the Stroke Register of Malmö [20], and the Swedish National Hospital Discharge Register [21], the latter for those who suffered from a stroke in hospitals outside of Malmö. In the Malmö stroke register, which has monitored stroke incidence in Malmö 1989–2010 [20], all stroke diagnoses were validated by review of medical records (63% of all stroke cases in this study), according to the WHO stroke definition. Computed tomography or magnetic resonance imaging of the brain, or autopsy had been done in all stroke cases classified as ischemic, intracerebral hemorrhage or subarachnoid hemorrhage. If no imaging had been performed the case was classified as unspecified stroke. From 2011 to 2016, all stroke cases were retrieved from the Swedish National Hospital Discharge register. International Classification of Diseases ICD-9 code 434 and the ICD-10 code I63 was used to identify incident cases of ischemic stroke in this register.

### Statistical analyses

Variables with a skewed distribution (C3, CRP and suPAR) were log-transformed. Baseline characteristics are reported separately for subjects without incident CE or ischemic stroke, subjects with only incident CE, subjects with only incident ischemic stroke, and subjects with both incident CE and ischemic stroke during follow up. Continuous data were described as means ± standard deviations (SD) or median (25–75%) for skewed distribution, and as proportions for categorical variables. Differences in the baseline characteristics of the study population according to incident disease status were tested using chi-square for categorical variables and ANOVA for continuous variables.

Analysis was conducted using Cox proportional hazards regression models to investigate associations between various risk factors and CE and ischemic stroke as endpoints. Hazard ratios (HRs) with 95% confidence intervals (CIs) were calculated using standardized values (Z scores) for the continuous variables and specified reference categories for the dichotomous variables. Follow-up time until the end, death, emigration or incident CE/ischemic stroke was used as time-scale. For the analysis conducted in MDCS, Model 1 was adjusted for age and sex. Model 2 was additionally adjusted for waist circumference, DM, smoking, systolic blood pressure, use of anti-hypertensive medication, ApoA1, and ApoB.

For the analysis in the MDC-CV, Model 1 was adjusted for age and sex. Model 2 was additionally adjusted for waist circumference, smoking, DM, systolic blood pressure, use of anti-hypertensive medications and LDL cholesterol.

The heterogeneity in the strength of association between CE and ischemic stroke was assessed using a modified method of Lunn-McNeil competing risks models using a data duplication method [22]. Briefly, this consists of duplicating the dataset, so that each individual appears in two strata. The failures (CE or ischemic stroke) are then sorted by strata, and a stratified Cox regression is performed, which thus allows the estimation of separate hazard ratios (HRs) for the two outcomes. The HRs (95% CI) obtained from this analysis is identical to the values obtained from the separate Cox regression in the original data set. Another analysis is then done with just one variable for the exposure of interest while rest of the model is kept the same. The likelihood ratio test is then conducted to compare the second model to the first one (with differential effects). P-values for the difference in association of a given exposure to the separate outcomes are derived from this likelihood ratio test, and the null hypothesis is that both outcomes are equally associated with the risk factors. Compared to the original Lunn–McNeil model, the modified version has events in both strata if the participants had developed both CE and ischemic stroke. The HRs obtained from this approach are identical to results from separate Cox models run for each outcome, which was verified in standard Cox regression models.

The proportional hazard assumptions were tested by incorporating the time-dependent effects of covariates, and also by calculating Schoenfeld residuals to inspect it visually. It was found that there was some deviation from the assumption for certain exposures. The analyses were, therefore, rerun for follow up time intervals before and after the median. However, visual inspection of the Schoenfeld residuals of the deviations and dividing up the follow up time indicated that the effect of this deviation on HRs was minor.

To further examine the robustness of the analysis, a sensitivity analysis was conducted after excluding those with both ischemic stroke and CE. Furthermore, as part of our sensitivity analysis, we stratified the ischemic stroke cases into those with and without atrial fibrillation (AF) before the ischemic stroke event. The analysis was repeated after excluding those with AF before ischemic stroke event.

Analyses were conducted using IBM SPSS Statistics version 24 (IBM Corp, Armonk, New York, USA) or STATA version 12.0 (StataCorp. 2011. Stata Statistical Software: Release 12. College Station, TX: StataCorp LP.). A *p* value of < 0.05 was regarded as statistically significant.

## Results

### Baseline characteristics

The baseline characteristics of the study population are summarized in Tables 1 and 2 for the MDCS and MDC-CV, respectively. In relation to the disease status, those who developed incident CE, ischemic stroke or both had higher cardiovascular risk factors as compared to those with no disease status in the MDCS (Table 1).

**Table 1:** Baseline characteristics in relation to incident ischemic stroke and incident CE status in the MDCS (*n* = 26,519)

	Population without CE or ischemic stroke (*n* = 21,594)	With only incident ischemic stroke (*n* = 1838)	With only incident CE (*n* = 2655)	With both incident CE and ischemic stroke (*n* = 432)	*p* value
Age (years)	57.23 (± 7.51)	61.65 (± 7.07)	61.20 (± 7.01)	62.93 (± 6.47)	< 0.001
Sex (men) n %	7491 (34.7)	864 (47)	1559 (58.7)	231 (53.5)	< 0.001
Waist (cm)	82.83 (± 14.68)	86.99 (± 12.88)	89.32 (± 12.67)	89.42 (± 12.29)	< 0.001
Diabetes n %	659 (3.1)	134 (7.3)	241 (9.1)	56 (13)	< 0.001
Smoking n %	5892 (27.3)	547 (29.8)	912 (34.4)	142 (32.9)	< 0.001
Systolic blood pressure (mmHg)	139.17 (± 19.40)	148.56 (± 20.61)	148.74 (± 20.19)	153.09 (± 21.06)	< 0.001
Anti-hypertensive medication n %	3140 (14.5)	437 (23.8)	691 (26)	146 (33.8)	< 0.001
ApoA1 (mg/dL)	158.50 (± 28.08)	155.10 (± 28.42)	148.78 (± 26.67)	150.09 (± 26.52)	< 0.001
ApoB (mg/dL)	105.30 (± 25.89)	111.73 (± 25.16)	116.47 (± 25.66)	115.56 (± 27.11)	< 0.001
Total leukocyte count (10^9^/L)	6.3 (± 1.64)	6.52 (± 1.74)	6.69 (± 1.77)	6.59 (± 1.72)	< 0.001
Lymphocyte count (10^9^/L)	1.94 (± 0.61)	1.97 (± 0.68)	1.99 (± 0.66)	2.00 (± 0.61)	< 0.001
Neutrophil count (10^9^/L)	3.85 (± 1.30)	4.02 (± 1.37)	4.15 (± 1.40)	4.05 (± 1.39)	< 0.001
Mixed cell count (10^9^/L)	0.51 (± 0.20)	0.53 (± 0.21)	0.55 (± 0.22)	0.55 (± 0.20)	< 0.001

Values expressed are means (± SD) or percentages unless specified elsewise

**Table 2:** Baseline characteristics in relation to incident ischemic stroke and incident CE status in the MDC-CV (*n* = 4795)

	Population without CE or ischemic stroke (*n* = 3935)	With only incident ischemic stroke (*n* = 320)	With only incident CE (*n* = 447)	With both CE and ischemic stroke (*n* = 93)	*p* value
Age (years)	57.14 (5.95)	59.93 (5.38)	59.64 (5.44)	61.40 (4.44)	< 0.001
Sex (men) n %	1450 (36.8)	147 (45.9)	266 (59.5)	51 (54.8)	< 0.001
Waist (cm)	82.47 (12.52)	85.91 (12.76)	88.44 (13.34)	89.23 (12.48)	< 0.001
Diabetes n %	120 (3)	30 (9.4)	42 (9.4)	12 (12.9)	< 0.001
Smoking n %	1003 (25.5)	97 (30.3)	137 (30.6)	32 (34.4)	0.002
Systolic blood pressure (mmHg)	139.74 (18.63)	146.57 (19.19)	148.06 (18.55)	154.13 (20.21)	< 0.001
Anti-hypertensive medication n %	538 (13.7)	76 (23.8)	97 (21.7)	31 (33.3)	< 0.001
LDL cholesterol (mmol/L)	4.14 (0.99)	4.21 (1.07)	4.30 (0.92)	4.39 (1.01)	0.001
Orosomucoid (g/L) 53 missing	0.70 (± 0.21)	0.73 (± 0.22)	0.73 (± 0.23)	0.79 (± 0.28)	< 0.001
Haptoglobin (g/L) 431 missing	1.30 (± 0.54)	1.37 (± 0.58)	1.38 (± 0.59)	1.42 (± 0.62)	0.002
Alpha1-antitrypsin (g/L) 80 missing	1.20 (± 0.27)	1.20 (± 0.30)	1.25 (± 0.29)	1.32 (± 0.32)	< 0.001
Ceruloplasmin (g/L) 288 missing	0.51 (± 0.12)	0.51 (± 0.13)	0.51 (± 0.11)	0.52 (± 0.11)	0.455
C3^a^ (g/L) 5 missing	1.48 (1.30–1.70)	1.52 (1.36–1.72)	1.55 (1.36–1.76)	1.54 (1.40–1.81)	0.002
CRP^a^ (mg/L) 161 missing	1.30 (0.60–2.60)	1.50 (0.70–3.40)	1.60 (0.80–3.30)	1.85 (0.98–3.33)	< 0.001
suPAR^a^ (ng/mL) 126 missing	2.79 (2.40–3.34)	3.04 (2.52–3.65)	2.88 (2.48–3.63)	3.13 (2.69–3.57)	< 0.001

Values expressed are means (± SD) or percentages unless specified elsewise

^a^Median [25%–75%]

Similarly, in the MDC-CV (Table 2) those with both incident CE and ischemic stroke showed relatively higher levels of inflammatory markers.

### Incident CE and ischemic stroke

In the MDCS, there were 2270 incident ischemic stroke events (mean follow-up time 19.43 ± 5.82 years) and 3087 incident CE events (mean follow-up time 19.33 ± 5.87 years) in the MDCS (Table 3). Both CE and ischemic stroke developed in 431 individuals, which, after exclusion resulted in 1838 incident ischemic stroke and 2656 incident CE events (Additional file 2: Table 1). In the MDC-CV, there were in total 320 and 447 incident cases of ischemic stroke (mean follow up 20.78 ± 5.69 years) and CE (mean follow up 20.68 ± 5.78 years), respectively, whereas 93 individuals had both incident CE and ischemic stroke (Table 2).

**Table 3:** Hazard ratios (95%CI) for incident ischemic stroke and incident CE in relation to risk factors in MDCS (*n* = 26,519)

		Incidence ischemic stroke (n = 2270)	Incidence coronary events (n = 3087)	P value for equal associations^d^
Age^e^	Model 1	2.01 (1.92–2.10)^c^	1.84 (1.77–1.91)^c^	0.003
	Model 2	1.84 (1.75–1.93)^c^	1.69 (1.62–1.76)^c^	0.011
Men	Model 1	1.56 (1.44–1.69)^c^	2.37 (2.20–2.54)^c^	< 0.001
	Model 2	1.35 (1.23–1.48)^c^	1.85 (1.70–1.98)^c^	< 0.001
≤ 9 years of schooling	Model 1	1.18 (1.07–1.30)^b^	1.42 (1.31–1.55)^c^	0.005
	Model 2	1.09 (0.99–1.20)	1.24 (1.14–1.35)^c^	0.040
Current Smoking	Model 1	1.56 (1.42–1.70)^c^	1.82 (1.67–1.96)^c^	0.010
	Model 2	1.62 (1.48–1.77)^c^	1.84 (1.70–1.98)^c^	0.038
Waist circumference^e^	Model 1	1.08 (1.06–1.10)^c^	1.08 (1.07–1.10)^c^	0.716
	Model 2	1.06 (1.03–1.09)^c^	1.07 (1.04–1.09)^c^	0.898
Systolic blood pressure ^e^	Model 1	1.31 (1.26–1.37)^c^	1.33 (1.28–1.38)^c^	0.618
	Model 2	1.27 (1.22–1.33)^c^	1.27 (1.22–1.32)^c^	0.973
Diabetes	Model 1	2.18 (1.88–2.53)^c^	2.49 (2.21–2.81)^c^	0.168
	Model 2	1.91 (1.64–2.22)^c^	2.06 (1.82–2.34)^c^	0.443
ApoA1^e,f^	Model 1	0.90 (0.86–0.94)^c^	0.77 (0.73–0.80)^c^	< 0.001
	Model 2	0.94 (0.90–0.98)^c^	0.81 (0.78–0.84)^c^	< 0.001
ApoB^e,f^	Model 1	1.12 (1.08–1.17)^c^	1.31 (1.27–1.36)^c^	< 0.001
	Model 2	1.06 (1.02–1.11)^b^	1.24 (1.19–1.28)^c^	< 0.001
Total leukocyte count^e^	Model 1	1.19 (1.14–1.23)^c^	1.28 (1.24–1.32)^c^	0.004
	Model 2	1.07 (1.03–1.12)^b^	1.11 (1.07–1.15)^c^	0.228
Lymphocyte count^e^	Model 1	1.10 (1.06–1.14)^c^	1.12 (1.09–1.15)^c^	0.342
	Model 2	1.03 (0.99–1.07)	1.02 (0.99–1.06)	0.753
Neutrophil count^e^	Model 1	1.17 (1.12–1.21)^c^	1.25 (1.21–1.29)^c^	0.007
	Model 2	1.07 (1.03–1.12)^b^	1.12 (1.08–1.16)^c^	0.154
Mixed cell count^e^	Model 1	1.05 (1.01–1.10)^b^	1.12 (1.08–1.16)^c^	0.026
	Model 2	1.00 (0.96–1.04)	1.04 (1.00–1.07)^a^	0.206

Model 1: Adjusted for age and sex

Model 2: Adjusted model for age, sex, waist circumference, diabetes, smoking, systolic blood pressure, use of anti-hypertensive medication, apolipoprotein A1 (ApoA1) and apolipoprotein B (ApoB)

^a^*p* < 0.05; ^b^*p* < 0.01; ^c^*p* < 0.001

^d^Null hypothesis for the *p-*value is that the variable has the same association with incident ischemic stroke and coronary events

^e^per 1 SD change in risk factor

^f^Adjusted for ApoA1 or ApoB

**Fig. 1:**
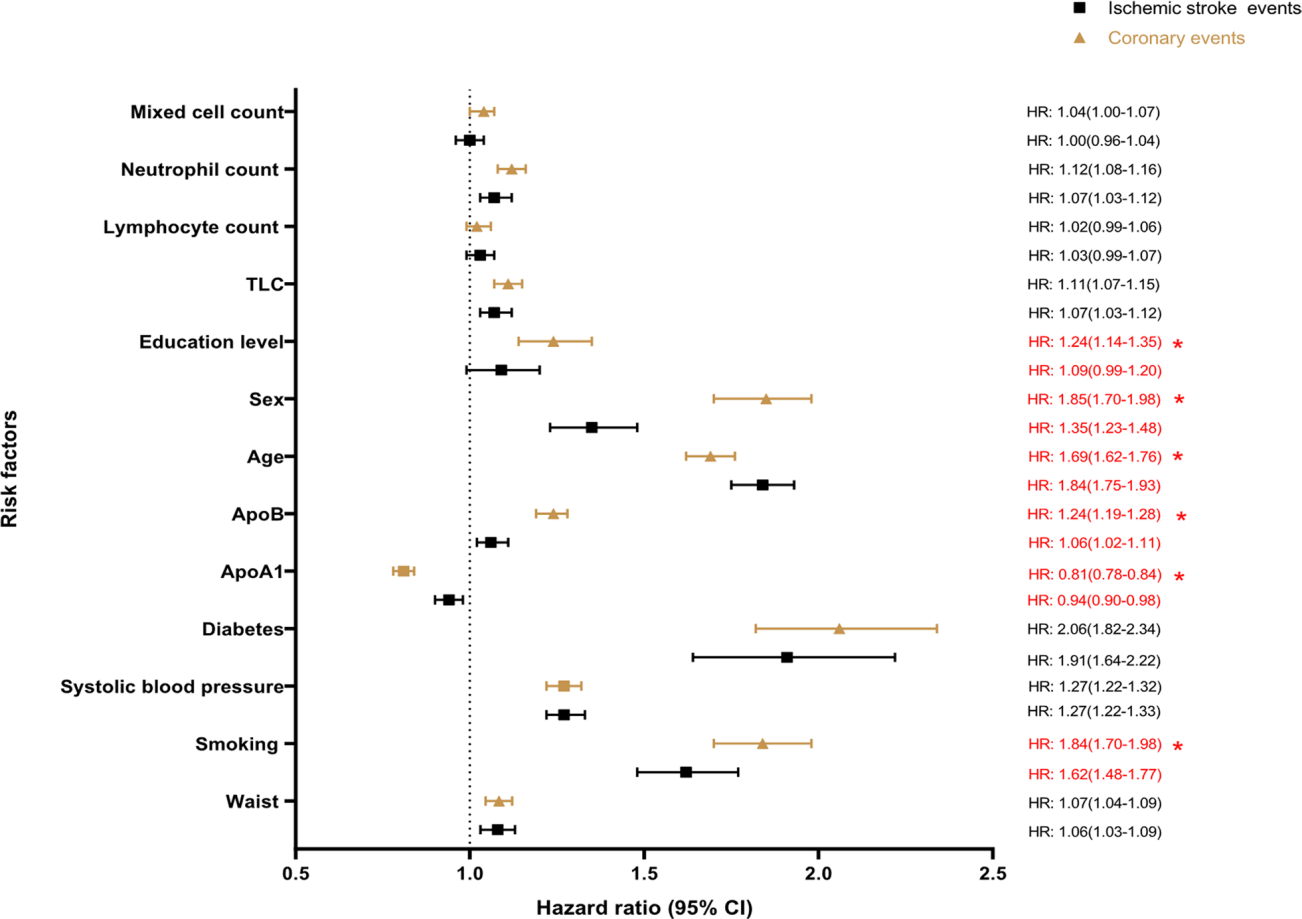
Hazard ratios (95% CI) for ischemic stroke and coronary events in relation to risk factors in MDCS

**Fig. 2:**
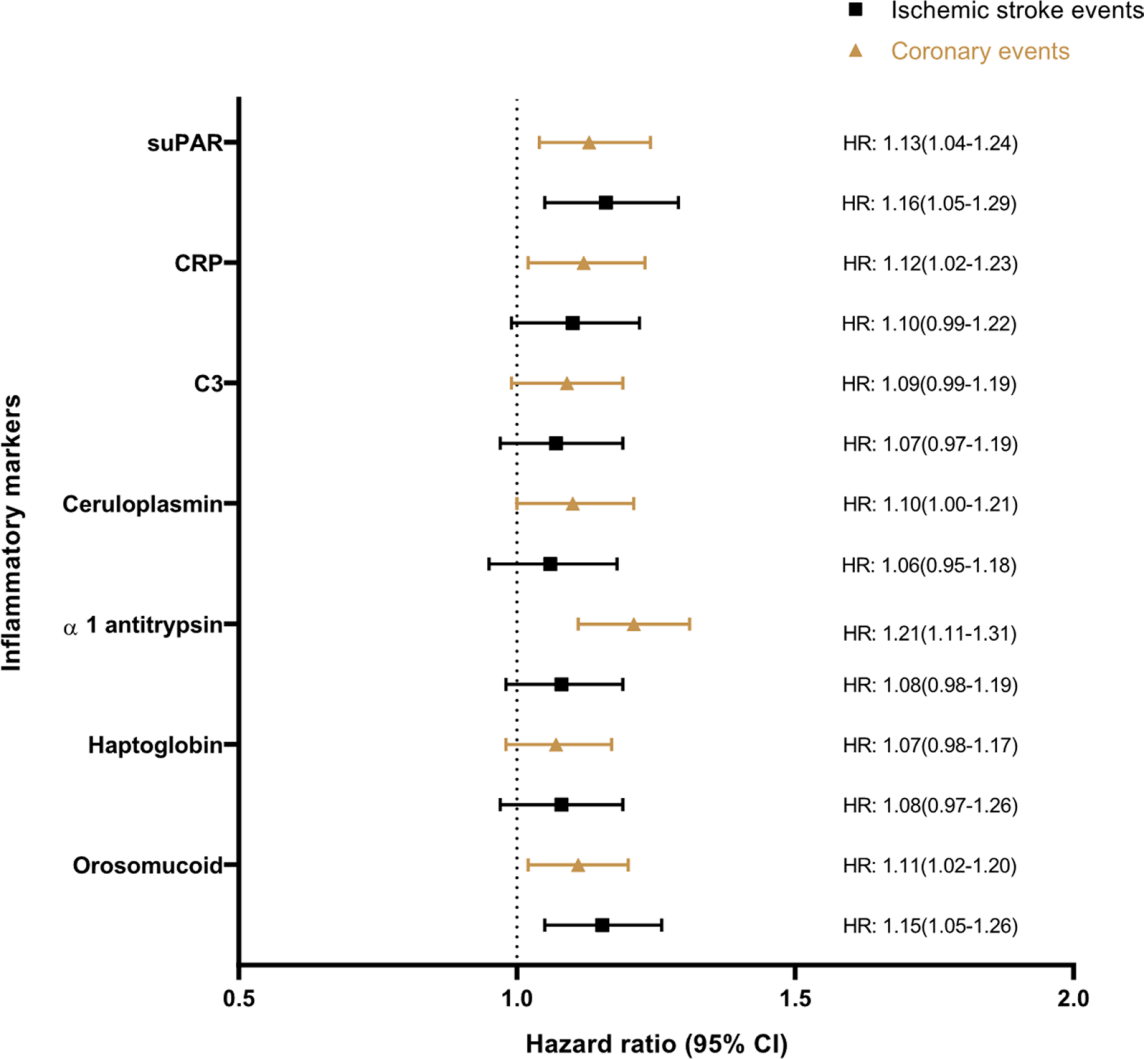
Hazard ratios (95% CI) for ischemic stroke and coronary events per 1SD change in inflammatory markers in MDC-CV

The age and sex adjusted and multivariable adjusted HRs for the multiple risk factors are shown in Table 3 and Fig. 1 for MDCS, and in Table 4 and Fig. 2 for MDC-CV. In Model 2, waist circumference, smoking, systolic blood pressure, DM, ApoB, age, sex, total leukocyte count, and neutrophil count were positively associated with risk of both CE and ischemic stroke. Schooling of ≤ 9 years was associated with an increased risk of incident CE but was not associated with incident ischemic stroke. ApoA1 levels were associated with reduced risk of both outcomes. In Model 2, lymphocyte count did not show significant association with either CE or ischemic stroke. Similar results were observed in the sensitivity analysis when participants with both CE and ischemic stroke were excluded (Additional file 2: Table 1).

**Table 4:** Hazard ratios (95%CI) for incident ischemic stroke and incident CE per 1 SD change in inflammatory markers in MDC-CV

	No. of subjects		Ischemic stroke		Coronary events		P value for equal associations^d^
			Events, n	HR (95%CI)	Events, n	HR (95%CI)	
Orosomucoid	4742	Model 1	408	1.22 (1.12–1.33)^c^	537	1.20 (1.11–1.29)^c^	0.735
		Model 2		1.15 (1.05–1.26)^b^		1.11 (1.02–1.20)^a^	0.858
Haptoglobin	4364	Model 1	368	1.17 (1.07–1.29)^b^	487	1.18 (1.09–1.28)^c^	0.961
		Model 2		1.08 (0.97–1.19)		1.07 (0.98–1.17)	0.906
Alpha1-antitrypsin	4715	Model 1	404	1.13 (1.03–1.24)^a^	529	1.25 (1.16–1.36)^c^	0.092
		Model 2		1.08 (0.98–1.19)		1.21 (1.11–1.31)^c^	0.093
Ceruloplasmin	4507	Model 1	383	1.09 (0.98–1.21)	502	1.14 (1.05–1.25)^b^	0.481
		Model 2		1.06 (0.95–1.18)		1.10 (1.00–1.21)^a^	0.567
C3	4790	Model 1	412	1.06 (1.01–1.11)^a^	540	1.07 (1.03–1.11)^b^	0.791
		Model 2		1.07 (0.97–1.19)		1.09 (0.99–1.19)	0.823
CRP	4634	Model 1	387	1.23 (1.11–1.36)^c^	507	1.26 (1.16–1.37)^c^	0.722
		Model 2		1.10 (0.99–1.22)		1.12 (1.02–1.23)^a^	0.758
suPAR	4669	Model 1	392	1.26 (1.16–1.38)^c^	506	1.23 (1.07–1.33)^c^	0.599
		Model 2		1.16 (1.05–1.29)^b^		1.13 (1.04–1.24)^b^	0.693

Model 1: Adjusted for age and sex

Model 2: Adjusted for age, sex, waist circumference, smoking, diabetes, systolic blood pressure, use of anti-hypertensive medication and LDL

^a^*p* < 0.05; ^b^*p* < 0.01; ^c^*p* < 0.001

^d^Null hypothesis for the *p *value is that the variable has the same association with incident ischemic stroke and coronary events

In the MDC-CV, the inflammatory markers were associated with higher risk of CE and ischemic stroke in Model 1. However, after adjusting for potential confounders, only orosomucoid, and suPAR were significantly associated with increased risk of ischemic stroke. On the other hand, orosomucoid, alpha1-antitrypsin, ceruloplasmin, CRP and suPAR were associated with incident CE after adjustment for confounders.

### Comparing risk factors for incident CE and ischemic stroke

Some important differences between the outcomes were found in relation to the baseline risk factors. Table 3 reports these data for the MDCS. In the final model, current smoking, ApoB, male sex and ≤ 9 years of schooling were preferentially associated with incident CE as compared to ischemic stroke (p for equal association = 0.038, < 0.001, < 0.001, and 0.040, respectively). ApoA1 was more protective for CE (HR_CE_ 0.81, CI 0.78–0.84) as compared to ischemic stroke (HR_st_ 0.94, CI 0.90–0.98) (p for equal association < 0.001). Age showed stronger association with ischemic stroke (HR_st_ 1.84, CI 1.75–1.93) as compared to CE (HR_CE_ 1.69, CI 1.62–1.76) (p for equal association = 0.011). Conversely, although some leukocytes were positively associated with both increased risk for CE and ischemic stroke, no difference in strength of association was observed for the two outcomes. Similar results were observed after excluding those both incident CE and ischemic stroke (Additional file 2: Table 1).

In Table 4, the associations of inflammatory markers in relation to incident CE and ischemic stroke are shown. Although most inflammatory markers were associated with both CE and ischemic stroke, no significant heterogeneity was found. Interestingly, in the sensitivity analysis (Additional file 2: Table 2) alpha1-antitrypsin showed a stronger association with CE after excluding participants with both CE and ischemic stroke. However, it was not significantly associated with risk of ischemic stroke.

After excluding ischemic strokes cases with AF (n = 503), in the MDCS, the difference in association was no longer significant for current smoking, age and education level. However, ApoA1, ApoB and male sex showed similar stronger association for CE (Additional file 2: Table 3). In the MDC-CV, after excluding those with AF, the results were essentially the same (Additional file 2: Table 4).

## Discussion

The main result of this study is that most cardiovascular risk factors show broadly similar associations with CE and ischemic stroke, but there are some differences in terms of the magnitude of associations. Current smoking, ApoA1, ApoB, male sex and education level showed stronger associations with CE whereas age was preferentially associated with ischemic stroke. However, in relation to inflammatory markers, no difference in association was observed. The results highlight the importance of looking at CE and ischemic stroke separately and, if possible, to avoid composite CVD endpoints in research studies.

The present study compared risk estimates for CE and ischemic stroke across a wide range of risk factors. Separating the composite endpoints of CVD and comparing the magnitudes of association provides better insight into the associations with various risk factors, and may lead to a better understanding of the underlying mechanism behind these associations. This may be useful in order to construct better risk stratification profiles [23].

The preferential association of lipids with CE rather than stroke observed in this study is in line with previous studies [24], and the stronger association of smoking, male sex and low education levels with CE further show that the risk factor profile for coronary disease does differ somewhat from ischemic stroke. As the etiology of ischemic stroke is heterogeneous and about 15% of cases are caused by large artery atherosclerosis [25] compared to myocardial infarctions, the differences in the risk factor patterns may reflect differences in risk factors between the ischemic stroke etiological subtypes.

One somewhat unexpected finding was that no difference was found for the association between systolic blood pressure and CE and ischemic stroke. Evidence from previous studies have shown that higher blood pressure is a greater risk factor for all-cause stroke than CE [26]. However, although hypertension is a risk factor for both ischemic and hemorrhagic stroke, high blood pressure is a greater risk for hemorrhagic than ischemic stroke, especially for intracerebral hemorrhage with non-lobar location [27]. The fact that the present analysis was restricted to ischemic stroke could likely explain why no significant difference between CE and ischemic stroke was observed.

Total and differential leukocyte count are markers of low grade inflammation and have been shown to be associated with both CE and stroke [28], 29. Previous findings from the same cohort showed that total leukocyte count and neutrophil count were significantly associated with ischemic stroke whereas lymphocyte count and mixed cell count were not [14], which is similar to our findings.

Although no difference in association was observed in relation to the inflammatory markers in the MDC-CV, they were predictive of increased risk for both CE and ischemic stroke suggesting a common pathway in terms of inflammation [30]. The role of inflammation has been shown to be central to atherosclerotic pathogenesis [31]. Hence, these inflammatory markers form part of the risk factor profile of both conditions.

There is limited epidemiological data exploring the differential association in CE and ischemic stroke with risk factors [11, 12]. The heterogeneity in the associations of risk factors in women was recently explored in a large study [32]. Results from our study showed that although most of the risk factors were positively associated with both incident CE and ischemic stroke, there is difference in the magnitude of association. Furthermore, studies have shown that stroke patients have a higher risk for CE [33, 34] and also, there is markedly increased risk for stroke after myocardial infarction [35], compared to subjects with no history of myocardial infarction. This can be attributed to the similar risk factor profile and in many cases an underlying atherosclerotic condition. The largely similar general risk factor profiles of CE and ischemic stroke also supports that many of the risk factors are shared between the ischemic stroke etiological subtypes, the largest groups being cardioembolism and small vessel occlusion, even if some differences also exist [36]. It is also interesting to note that when analysis was repeated for ischemic stroke and CE with no history of AF in our study, ApoB association remained stronger for CE, reflecting a difference in relation to atherosclerotic strokes and etiologies.

There are many strengths of the study. It is a large prospective cohort study with a long follow up period. The number of end-points and statistical power was high. The modified Lunn-McNeil method allowed us to have a direct comparison of risk factors for both CE and ischemic stroke within the same population and to give a quantitative measure of the strength of association. The endpoints were retrieved from high quality registers. Approximately 63% of the ischemic stroke cases were validated using hospital records. We were able to classify the incident stroke cases into the ischemic subtype, which is important since different stroke subtypes are associated with different risk factors [37]. However, while most ischemic stroke cases were validated, there is still a low risk for misclassification of stroke outcomes.

A few limitations of the study need to be considered. The risk factors were measured once at baseline, and many of these would have changed over the follow-up period. However, in that case our findings would present an underestimated risk, and bias the results towards the null, both for ischemic stroke and CE. Another thing to consider is correction for multiple testing which we did not carry out. It should be used with caution as use of multiple adjustment implies that interpretation of an association is based on the number of tests performed rather than what is demonstrated by the data [38]. Moreover, correction for multiple testing is a restrictive approach and can increase the risk of type II error [39] which we wanted to avoid. Since this is an observational study, confounding is a main concern. We adjusted the analysis for several potential confounders. Nevertheless, residual confounding cannot be completely ruled out. In addition, the study cohort consisted primarily of individuals of European descent, and thus the generalizability of the results might be limited.

In conclusion, traditional and inflammatory risk factors have broadly similar associations in relation to incident CE and ischemic stroke. However, the magnitude of association differs for some risk factors with the two outcomes, emphasizing the distinct biology of different vascular beds. This highlights the importance of looking at CE and ischemic stroke, two manifestations of CVD, separately, and if possible, to avoid composite CVD endpoints in research studies.

## Supplementary Information

**Additional file 1.** Study population flow chart.

**Additional file 2.** Supplementary results.

## Data Availability

The authors do not own the data underlying this study. The data are owned by Lund University, and the approval for research from this database is obtained through the Malmö Diet and Cancer Study (MDCS) steering committee. The data cannot be made publicly available without ethical approval. Data are available upon request for interested researchers by applying to MDCS steering committee. Please email (Anders.Dahlin@med.lu.se) with requests for the data.

## Abbreviations

AF: Atrial fibrillation
ApoA1: Apolipoprotein A1
ApoB: Apolipoprotein B
CI: Confidence intervals
CE: Coronary events
CRP: C-reactive protein
CVD: Cardiovascular diseases
DM: Diabetes mellitus
ICD: International Classification of Diseases
MDCS: Malmö diet and cancer study
MDCS-CV: Malmö Diet and Cancer Cardiovascular cohort
suPAR: Soluble urokinase plasminogen activator receptor
