# Supplementary material for: Comparison of risk factors for ischemic stroke and coronary events in a population-based cohort
Source: BMC Cardiovasc Disord. 2021 Nov 12;21:536. doi: 10.1186/s12872-021-02344-4 (PMC8588679; doi:10.1186/s12872-021-02344-4)
Supplement: Supplementary file 2 — Additional file 2. Supplementary results. [file 12872_2021_2344_MOESM2_ESM.docx]

**Supplementary Table 1: Hazard ratios (95%CI) for ischemic stroke and coronary events in relation to risk factors in MDCS after excluding those with both events *(n=26,087):***

|  |  | Incidence ischemic stroke  *n=1838* | Incidence coronary events  *n=2655* | P value for equal associations ^d^ |
| --- | --- | --- | --- | --- |
| Age ^e^ | Model 1 | 1.96(1.86-2.06) ^c^ | 1.79(1.72-1.86) ^c^ | <0.001 |
|  | Model 2 | 1.81(1.71-1.91) ^c^ | 1.65(1.58-1.72) ^c^ | 0.011 |
| Men | Model 1 | 1.49(1.36-1.64) ^c^ | 2.46(2.28-2.66) ^c^ | <0.001 |
|  | Model 2 | 1.32(1.19-1.46) ^c^ | 1.92(1.76-2.10) ^c^ | <0.001 |
| Current Smoking | Model 1 | 1.50(1.36-1.66) ^c^ | 1.82(1.68-1.98) ^c^ | 0.003 |
|  | Model 2 | 1.55(1.40-1.72) ^c^ | 1.82(1.67-1.98) ^c^ | 0.018 |
| ≤ 9 years of schooling | Model 1 | 1.17(1.05-1.30) ^a^ | 1.45(1.32-1.58) ^c^ | 0.003 |
|  | Model 2 | 1.09(0.98-1.21) | 1.27(1.16-1.39) ^c^ | 0.029 |
| Waist circumference ^e^ | Model 1 | 1.08(1.05-1.10) ^c^ | 1.08(1.07-1.10) ^c^ | 0.665 |
|  | Model 2 | 1.06(1.03-1.09) ^c^ | 1.06(1.03-1.09) ^c^ | 0.909 |
| Systolic blood pressure ^e^ | Model 1 | 1.28(1.22-1.34) ^c^ | 1.31(1.26-1.36) ^c^ | 0.414 |
|  | Model 2 | 1.25(1.19-1.31) ^c^ | 1.26(1.21-1.31) ^c^ | 0.890 |
| Diabetes | Model 1 | 2.06(1.67-2.54) ^c^ | 2.84(2.44-3.30) ^c^ | 0.013 |
|  | Model 2 | 1.81(1.47-2.24) ^c^ | 2.31(1.98-2.69) ^c^ | 0.068 |
| ApoA1 ^e f^ | Model 1 | 0.93(0.88-0.98) ^b^ | 0.76(0.73-0.80) ^c^ | <0.001 |
|  | Model 2 | 0.96(0.91_1.01) | 0.80(0.77-0.84) ^c^ | <0.001 |
| ApoB ^e f^ | Model 1 | 1.10(1.05-1.15) ^c^ | 1.32(1.27-1.37) ^c^ | <0.001 |
|  | Model 2 | 1.05(1.00-1.10) | 1.25(1.21-1.30) ^c^ | <0.001 |
| Total leukocyte count ^e^ | Model 1 | 1.18(1.13-1.24) ^c^ | 1.29(1.25-1.34) ^c^ | 0.002 |
|  | Model 2 | 1.09(1.03-1.14) ^b^ | 1.13(1.08-1.17) ^c^ | 0.247 |
| Lymphocyte count ^e^ | Model 1 | 1.09(1.05-1.14) ^c^ | 1.12(1.09-1.15) ^c^ | 0.288 |
|  | Model 2 | 1.03(0.99-1.08) | 1.02(0.98-1.08) | 0.705 |
| Neutrophil count ^e^ | Model 1 | 1.23(1.16-1.30) ^c^ | 1.37(1.30-1.43) ^c^ | 0.004 |
|  | Model 2 | 1.11(1.05-1.19) ^a^ | 1.18(1.12-1.24) ^c^ | 0.166 |
| Mixed cell count ^e^ | Model 1 | 1.06(1.01-1.11) ^a^ | 1.14(1.09-1.18) ^c^ | 0.017 |
|  | Model 2 | 1.01(0.96-1.06) | 1.05(1.01-1.09) ^b^ | 0.176 |

Model 1: Adjusted for age and sex

Model 2: Adjusted model for age, sex, waist circumference, diabetes, smoking, systolic blood pressure, use of anti-hypertensive medication, apolipoprotein A1 (ApoA1) and apolipoprotein B (ApoB)

^a^ *p*<0.05, ^b^ *p*<0.01, ^c^ *p*<0.001

^d^ Null hypothesis for the *p-*value is that the variable has the same association with incident ischemic stroke and coronary events.

^e^ per 1 SD change in risk factors ^f^ Adjusted for ApoA1 or ApoB

**Supplementary Table 2: Hazard ratios (95%CI) for ischemic stroke and coronary events per 1 SD change in inflammatory markers in MDC-CV after excluding those with both events:**

|  | No. of subjects |  | Ischemic stroke | | Coronary events | | P value for equal associations ^d^ |
| --- | --- | --- | --- | --- | --- | --- | --- |
|  |  |  | **Events, n** | **HR(95%CI)** | **Events, n** | **HR(95%CI)** |  |
| Orosomucoid | 4649 | Model 1 | 327 | 1.17(1.06-1.30) ^c^ | 464 | 1.17(1.08-1.27) ^c^ | 0.962 |
|  |  | Model 2 |  | 1.11(0.99-1.23) |  | 1.09(0.99-1.19) | 0.804 |
| Haptoglobin | 4284 | Model 1 | 297 | 1.16(1.04-1.30) ^b^ | 416 | 1.17(1.07-1.28) ^c^ | 0.898 |
|  |  | Model 2 |  | 1.08(0.96-1.22) |  | 1.07(0.97-1.18) | 0.909 |
| Alpha 1- anti trypsin | 4624 | Model 1 | 325 | 1.03(0.92-1.15) | 452 | 1.24(1.14-1.35) ^c^ | 0.018 |
|  |  | Model 2 |  | 0.99(0.88-1.10) |  | 1.19(1.08-1.30) ^c^ | 0.017 |
| Ceruloplasmin | 4422 | Model 1 | 309 | 1.05(0.93-1.18) | 427 | 1.33(1.02-1.25) ^b^ | 0.324 |
|  |  | Model 2 |  | 1.02(0.89-1.15) |  | 1.09(0.98-1.21) | 0.411 |
| C3 | 4697 | Model 1 | 331 | 1.05(0.99-1.12) | 468 | 1.07(1.03-1.12) ^b^ | 0.591 |
|  |  | Model 2 |  | 1.03(0.92-1.19) |  | 1.08(0.98-1.20) | 0.688 |
| CRP | 4548 | Model 1 | 312 | 1.20(1.07-1.34) ^b^ | 442 | 1.26(1.15-1.38) ^c^ | 0.505 |
|  |  | Model 2 |  | 1.09(0.97-1.23) |  | 1.13(1.02-1.25) ^b^ | 0.668 |
| suPAR | 4582 | Model 1 | 315 | 1.24(1.12-1.37) ^c^ | 440 | 1.22(1.12-1.32) ^c^ | 0.809 |
|  |  | Model 2 |  | 1.15(1.02-1.28) ^a^ |  | 1.12(1.02-1.24) ^a^ | 0.731 |

Model 1: Adjusted for age and sex

Model 2: Adjusted for age, sex, waist circumference, diabetes, smoking, systolic blood pressure, use of anti-hypertensive medication and LDL cholesterol

^a^ *p*<0.05, ^b^ *p*<0.01, ^c^ *p*<0.001

^d^ Null hypothesis for the *p-*value is that the variable has the same association with incident ischemic stroke and coronary events.

**Supplementary Table 3: Hazard ratios (95%CI) for ischemic stroke and coronary events in relation to risk factors in MDCS after excluding those with atrial fibrillation before ischemic stroke *(n=26,016):***

|  |  | Incidence ischemic stroke  *n=1767* | Incidence coronary events *n=2972* | P value for equal associations ^d^ |
| --- | --- | --- | --- | --- |
| Waist circumference ^e^ | Model 1 | 1.08( 1.05-1.10) ^c^ | 1.08(1.07-1.10)^c^ | 0.425 |
|  | Model 2 | 1.05(1.01-1.09) ^a^ | 1.07(1.04-1.09)^c^ | 0.445 |
| Current Smoking | Model 1 | 1.67(1.51-1.85) ^c^ | 1.82(1.68-1.96)^c^ | 0.199 |
|  | Model 2 | 1.72(1.55-1.90) ^c^ | 1.83(1.69-1.98)^c^ | 0.325 |
| Systolic blood pressure ^e^ | Model 1 | 1.32(1.26-1.38) ^c^ | 1.34(1.29-1.39)^c^ | 0.689 |
|  | Model 2 | 1.28(1.23-1.35) ^c^ | 1.28(1.23–1.32)^c^ | 0.755 |
| Diabetes | Model 1 | 2.33(1.98–2.75) ^c^ | 2.58(2.29–2.92)^c^ | 0.333 |
|  | Model 2 | 2.07(1.75–2.44) ^c^ | 2.12(1.87–2.40)^c^ | 0.815 |
| ApoA1 ^e f^ | Model 1 | 0.89(0.84-0.93) ^c^ | 0.76(0.73-0.80) ^c^ | <0.001 |
|  | Model 2 | 0.92(0.88-0.97) ^a^ | 0.81(0.77-0.84) ^c^ | <0.001 |
| ApoB ^e f^ | Model 1 | 1.16(1.10-1.21) ^c^ | 1.31(1.27-1.36) ^c^ | <0.001 |
|  | Model 2 | 1.09(1.04-1.15) ^c^ | 1.24(1.19-1.28) ^c^ | <0.001 |
| Age ^e^ | Model 1 | 1.87(1.78-1.97) ^c^ | 1.83(1.76-1.90) ^c^ | 0.509 |
|  | Model 2 | 1.71(0.62-1.81) ^c^ | 1.68(1.61-1.75) ^c^ | 0.523 |
| Men | Model 1 | 1.56(1.42-1.71) ^c^ | 2.39(2.22-2.57) ^c^ | <0.001 |
|  | Model 2 | 1.35(1.21-1.50) ^c^ | 1.86(1.72-2.02) ^c^ | <0.001 |
| ≤ 9 years of schooling | Model 1 | 1.24(1.11-1.38) ^c^ | 1.42(1.30-1.55) ^c^ | 0.054 |
|  | Model 2 | 1.13(1.01-1.26) ^a^ | 1.24(1.14-1.35) ^c^ | 0.184 |
| Total leukocyte count ^e^ | Model 1 | 1.21(1.16-1.27) ^c^ | 1.29(1.25-1.34) ^c^ | 0.018 |
|  | Model 2 | 1.08(1.03-1-14) ^b^ | 1.12(1.08-1.17) ^c^ | 0.220 |
| Lymphocyte count ^e^ | Model 1 | 1.11(1.07-1.15) ^c^ | 1.14(1.11-1.17) ^c^ | 0.289 |
|  | Model 2 | 1.02(0.98-1.07) | 1.03(0.99-1.06) | 0.967 |
| Neutrophil count ^e^ | Model 1 | 1.19(1.14-1.24) ^c^ | 1.26(1.22-1.30) ^c^ | 0.042 |
|  | Model 2 | 1.09(1.04-1.14) ^b^ | 1.13(1.09-1.17) ^c^ | 0.220 |
| Mixed cell count ^e^ | Model 1 | 1.05(1.01-1.10) ^a^ | 1.13(1.09-1.16) ^c^ | 0.018 |
|  | Model 2 | 1.00(0.95-1.04) | 1.05(1.01-1.08)^a^ | 0.106 |

Model 1: Adjusted for age and sex

Model 2: Adjusted model for age, sex, waist circumference, diabetes, smoking, systolic blood pressure, use of anti-hypertensive medication, apolipoprotein A1 (ApoA1) and apolipoprotein B (ApoB)

^a^ *p*<0.05, ^b^ *p*<0.01, ^c^ *p*<0.001

^d^ Null hypothesis for the *p-*value is that the variable has the same association with incident ischemic stroke and coronary events.

^e^ per 1 SD change in risk factors ^f^ Adjusted for ApoA1 or ApoB

**Supplementary Table 4: Hazard ratios (95%CI) for ischemic stroke and coronary events per 1 SD change in inflammatory markers in MDC-CV after excluding those with atrial fibrillation before ischemic stroke:**

|  | No. of subjects |  | Ischemic stroke | | Coronary events | | P value for equal associations ^d^ |
| --- | --- | --- | --- | --- | --- | --- | --- |
|  |  |  | **Events, n** | **HR(95%CI)** | **Events, n** | **HR(95%CI)** |  |
| Orosomucoid | 4656 | Model 1 | 322 | 1.25(1.14-1.37) ^c^ | 512 | 1.18(1.09-1.27) ^c^ | 0.375 |
|  |  | Model 2 |  | 1.18(1.07-1.31) ^b^ |  | 1.10(1.01-1.19) ^a^ | 0.263 |
| Haptoglobin | 4289 | Model 1 | 293 | 1.19(1.07-1.33) ^b^ | 468 | 1.16(1.07-1.27) ^c^ | 0.716 |
|  |  | Model 2 |  | 1.10(0.98-1.23) |  | 1.06(0.96-1.16) | 0.571 |
| α1-anti trypsin | 4631 | Model 1 | 320 | 1.11(1.00-1.23) | 505 | 1.24(1.15-1.35) ^c^ | 0.105 |
|  |  | Model 2 |  | 1.07(0.96-1.20) |  | 1.20(1.10-1.30) ^c^ | 0.109 |
| Ceruloplasmin | 4428 | Model 1 | 304 | 1.06(0.93-1.19) | 480 | 1.13(1.03-1.24) ^b^ | 0.373 |
|  |  | Model 2 |  | 1.03(0.91-1.17) |  | 1.09(0.99-1.20) | 0.439 |
| C3 | 4703 | Model 1 | 325 | 1.17(1.06-1.30) ^b^ | 515 | 1.20(1.11-1.30) ^c^ | 0.779 |
|  |  | Model 2 |  | 1.10(0.98-1.24) |  | 1.09(0.99-1.19) | 0.858 |
| CRP | 4551 | Model 1 | 304 | 1.24(1.11-1.39) ^c^ | 484 | 1.26(1.15-1.38) ^c^ | 0.852 |
|  |  | Model 2 |  | 1.12(0.99-1.26) |  | 1.12(1.02-1.23) ^a^ | 0.970 |
| suPAR | 4585 | Model 1 | 308 | 1.28(1.16-1.41) ^c^ | 482 | 1.23(1.13-1.33) ^c^ | 0.484 |
|  |  | Model 2 |  | 1.19(1.07-1.33) ^b^ |  | 1.14(1.04-1.25) ^b^ | 0.527 |

Model 1: Adjusted for age and sex

Model 2: Adjusted for age, sex, waist circumference, diabetes, smoking, systolic blood pressure, use of anti-hypertensive medication and LDL cholesterol

^a^ *p*<0.05, ^b^ *p*<0.01, ^c^ *p*<0.001

^d^ Null hypothesis for the *p-*value is that the variable has the same association with incident ischemic stroke and coronary events.
